# Redox Regulation and Oxidative Stress in Mammalian Oocytes and Embryos Developed *In Vivo* and *In Vitro*

**DOI:** 10.3390/ijerph182111374

**Published:** 2021-10-29

**Authors:** Madeleine L. M. Hardy, Margot L. Day, Michael B. Morris

**Affiliations:** Discipline of Physiology, School of Medical Sciences, Faculty of Medicine and Health, University of Sydney, Sydney 2006, Australia; mhar7073@uni.sydney.edu.au

**Keywords:** redox, ROS, embryo, oocyte, antioxidants, assisted reproductive technology, transgenerational effects

## Abstract

Oocytes and preimplantation embryos require careful regulation of the redox environment for optimal development both *in vivo* and *in vitro*. Reactive oxygen species (ROS) are generated throughout development as a result of cellular metabolism and enzyme reactions. ROS production can result in (i) oxidative eustress, where ROS are helpful signalling molecules with beneficial physiological functions and where the redox state of the cell is maintained within homeostatic range by a closely coupled system of antioxidants and antioxidant enzymes, or (ii) oxidative distress, where excess ROS are deleterious and impair normal cellular function. *in vitro* culture of embryos exacerbates ROS production due to a range of issues including culture-medium composition and laboratory culture conditions. This increase in ROS can be detrimental not only to assisted reproductive success rates but can also result in epigenetic and genetic changes in the embryo, resulting in transgenerational effects. This review examines the effects of oxidative stress in the oocyte and preimplantation embryo in both the *in vivo* and *in vitro* environment, identifies mechanisms responsible for oxidative stress in the oocyte/embryo in culture and approaches to reduce these problems, and briefly examines the potential impacts on future generations.

## 1. Introduction

Preimplantation embryo development requires tight regulation of molecular and physiological processes for optimal blastocyst formation, hatching and implantation. One set of mechanisms that need careful balance in these early stages of development is redox control within the oocyte and embryo, and the surrounding maternal environment [1,2]. The redox state of a cell depends on the ratio of oxidised and reduced molecules [3] and redox homeostasis (i.e., oxidative eustress) helps maintain normal cellular function [4,5,6].

**Figure 1:**
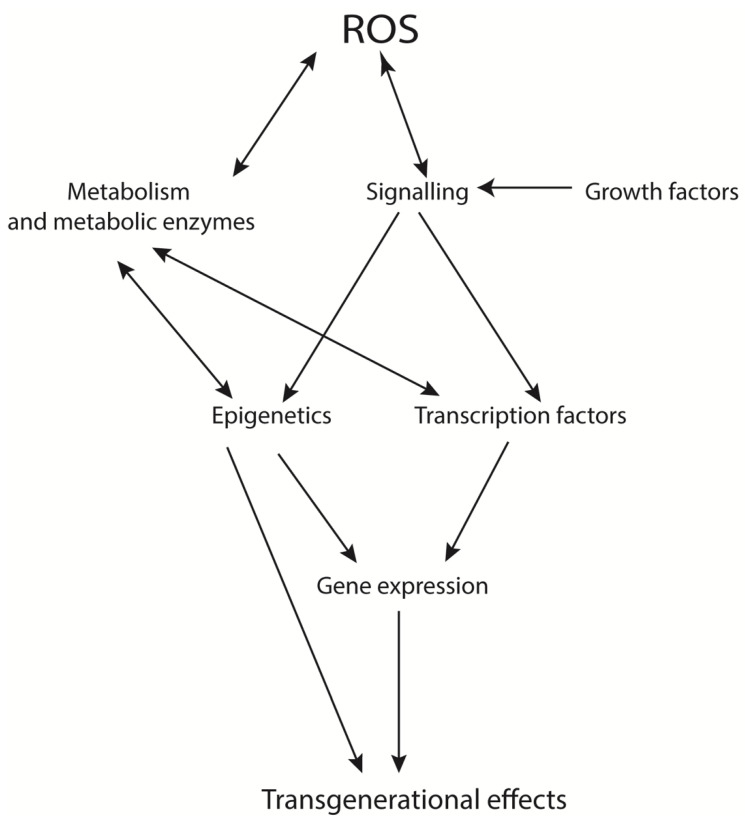
Effects of ROS on cellular function. ROS production has a multitude of impacts on cellular function and can act in both a beneficial and deleterious manner. ROS are produced as a ‘by-product’ of cellular metabolism and as a result of various cell-signalling pathways, including many activated by growth factors. They can also directly affect the activity of signalling pathway components and metabolic enzymes, leading to changes in cellular metabolic profile and energy usage. In turn, these changes in metabolism and signalling can lead to changes in the epigenetic landscape and the activity of transcription factors, altering gene expression. Since these events directly and indirectly affect the reproductive system, transgenerational effects resulting from normal and aberrant ROS production can and do occur.

Reactive oxygen species (ROS) are generated by a variety of cellular metabolic activities and, in particular, as a by-product of ATP generation mediated by mitochondrial respiration [7,8]. However, either excessive accumulation of ROS or highly reduced conditions upsets redox homeostasis, results in oxidative distress and, in the embryo, acts to impair development by a variety of mechanisms (Figure 1) [1,4,5,6,9,10,11]. The sensitivity of the oocyte and preimplantation embryo to oxidative stress also presents challenges for *in vitro* assisted reproductive technologies (ART), including oocyte maturation, fertilisation, and embryo culture. Redox imbalance during early development can also result in transgenerational effects to the immediate offspring and later generations [12,13,14].

This review examines the effects of oxidative stress in the oocyte and preimplantation embryo in both the *in vivo* and *in vitro* environment, identifies mechanisms responsible for oxidative stress that affect the current oocyte/embryo and effects on future generations, as well as approaches to reduce these problems.

## 2. Cellular ROS

ROS is a term used to describe oxygen-containing molecules that are highly reactive and electron-accepting [15]. Due to the fact the 2 lone electrons in the outer-most orbital of molecular oxygen, O_2_, have the same spin quantum number, it can only accept one electron at a time as it is reduced to H_2_O [8]. This results in the production of a series of intermediates, namely the free-radical superoxide anion (O_2_·^−^), non-radical hydrogen peroxide (H_2_O_2_), and the free-radical hydroxyl ion (OH·) [8]. (The formation of highly reactive singlet oxygen, ^1^O_2_, an electronically excited form of O_2_, and its conversion to the powerful oxidising agent ozone, O_3_, also occurs in animals. For a review, see Ref. [16]).

ROS production is ubiquitous in cells and, under conditions of oxidative eustress, ROS are now known to play an increasingly large number of roles in normal organismal physiology [5]. However, supraphysiological concentrations of ROS—i.e., the excessive production of ROS that cannot be counteracted by the cell’s natural antioxidant systems [17]—results in oxidative distress [4]. This oxidative stress can lead to a large variety of cellular-mechanism dysfunctions culminating, for example, in growth arrest and premature cell death [18,19,20,21].

### 2.1. Sources of Cellular ROS

**Figure 2:**
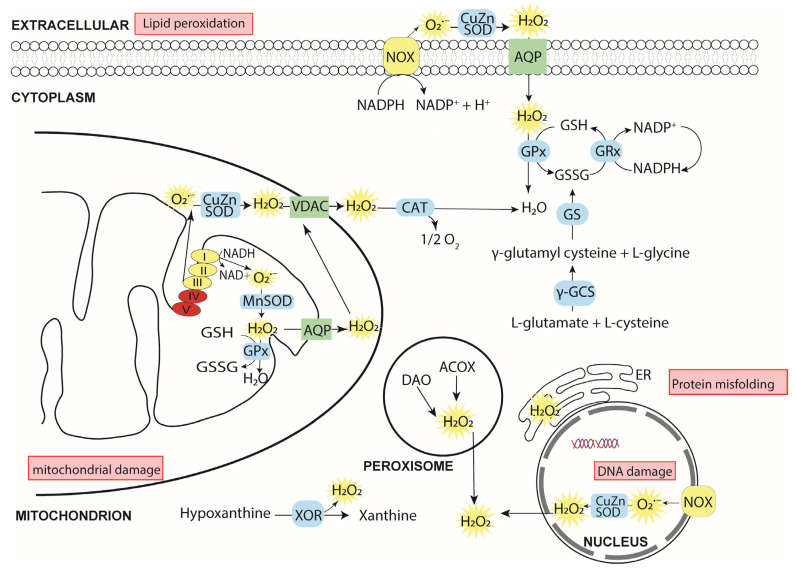
Cellular sources of ROS and antioxidants, and the effects of oxidative distress. Numerous cellular enzymes (yellow discs) produce ROS (shown in yellow sunbursts), whose homeostatic concentrations are controlled by a comprehensive system of antioxidants (only GSH is shown here) and antioxidant enzymes (blue discs) [37]. Principal sources are NOX, Complexes I–III of the mitochondrial electron transport chain due primarily to electron leakage [8,26], and to various enzymes (not shown), such as proline oxidase, which are coupled to Complex II [8,26]. The principal ROS signalling molecule is H_2_O_2_ (physiological concentration ≈1–10 nM [8,26]), which can penetrate membranes directly or (more efficiently) by transmembrane transporters (green disks). Oxidative distress occurs when the antioxidant system cannot maintain homeostatic concentrations of ROS, which can lead to a range of cellular dysfunctions (red boxes), frequently resulting in growth arrest and apoptosis. ACOX, acyl coenzyme A oxidase; AQP, aquaporin; CAT, catalase, CuZnSOD, copper–zinc superoxide dismutase; DAO, diamine oxidase; γ-GCS, γ-glutamylcysteine synthetase; GPx, glutathione peroxidase; GRx, glutathione reductase; GS, glutathione synthetase; MnSOD, manganese superoxide dismutase; NOX, NADPH oxidase; VDAC, voltage-dependent anion channel; XOR, xanthine oxidoreductase.

ROS are generated in many ways at physiologically relevant rates including: 

(i) Directly by enzyme-catalysed reactions. There are over 40 enzymes that generate O_2_·^−^/H_2_O_2_ (see Table 1 [4]) chief among them the NOX family of multi-subunit NADPH oxidases, the transmembrane components of which are responsible for transporting electrons across biological membranes: The oxidation of NADPH (to NADP^+^ and H^+^) on one side of the membrane results in concerted reduction of O_2_ to O_2_·^−^ (or H_2_O_2_) on the other [21] (Figure 2).

In addition to NOX, which are found principally in the plasma, nuclear and endoplasmic reticulum (ER) membranes, peroxisomes and lysosomes are major generators of ROS [18,20]. ROS are also produced in the plasma membrane following oxidation of arachidonic acid by cyclooxygenase and lipoxygenase [22]. Other sources include ROS production from amino-acid metabolism and the reduction of hypoxanthine to xanthine by xanthine oxidoreductase (XOR), which generates O_2_·^−^ [23] (Figure 2). 

(ii) As a ‘by-product’ of electron transport chain (ETC) flux. 0.12–2% of the O_2_ consumed by a cell *in vitro* is converted to O_2_·^−^ in the ETC [7,8,24,25,26], though the values *in vivo* are likely to be lower [8,26]. ETC-generated O_2_·^−^ can spontaneously dismutate to H_2_O_2_ but the rate is much slower than in the presence of physiological concentrations of mitochondrial superoxide dismutase (MnSOD) [26,27]. As a result, the concentration of O_2_·^−^ in mitochondria is as little as 10–200 pM [28], while physiologically relevant concentrations of H_2_O_2_ are maintained in the low nanomolar range (roughly 1–10 nM) [4].

There are at least 11 sites in the ETC where O_2_·^−^/H_2_O_2_ are produced. The principle sites are Complexes I–III where, in certain cellular states, generation can also occur by reverse electron transport [24,26]. Production can also occur via functional and physical interactions of enzymes, such as proline oxidase (POX), with ETC complexes [29]. For example, POX itself, which oxidises proline to pyrroline-5-carboxylate (P5C) doesn’t produce ROS but rather directs high-energy electrons into the ETC in the form of FADH_2_ through its coupling to Complex II [29,30,31,32]. Overexpression of POX and/or its increased activity in the presence of proline results in an acute burst of ROS via the ETC [8,26,29,31,33,34,35].

The balance of generation of ROS from mitochondrial and non-mitochondrial sources will depend on the cell type as well as its metabolic status at the time. In resting mouse skeletal muscle-derived myoblasts, there is roughly equal generation of H_2_O_2_ from each (~45% from the ETC and ~40% from NOX) [36]. 

### 2.2. The (Patho)Physiological Roles of ROS

#### 2.2.1. Oxidative Eustress

Although once deemed a toxic by-product of aerobic respiration that cells must remove in order to maintain normal function, the physiological roles of ROS have, more recently, come to the fore [4,20,38]. Levels of ROS generated from various enzymes and adventitious production from the ETC are held within homeostatic concentration ranges by a closely coupled system of antioxidants (e.g., glutathione (GSH), thioredoxin (Trx), vitamins C and E) and redox-regulating enzymes (Figure 2) [37,39,40]. This close coupling of generation and removal is exploited by cells in variety of ways: (i) Highly reactive ROS (in particular, H_2_O_2_) act as second messengers to modulate the activity of a variety of cellular macromolecules, including metabolic and signalling-pathway enzymes and components of cytoskeletal networks [4,41,42]. (ii) Growth factors and other molecules can stimulate signalling which generate ROS to initiate cellular response to environmental cues [43,44,45,46]. Collectively, ROS control a pleiotropic range of homeostatic cellular responses including aspects of the hypoxic response, stress response, antioxidant response, autophagy and metabolic adaptation [4,8,26,42], all of which are important for normal oocyte maturation and preimplantation embryo development. In particular, metabolic adaptation includes negative feedback by ETC-generated H_2_O_2_ to fine-tune control of mitochondrial respiration, the balance of molecular sources (e.g., fatty acids, amino acids and carbohydrates) that feed into the TCA cycle and ETC, and the balance of ATP production accorded to anaerobic and aerobic metabolism [4,8,26], and these switches occur at critical times in early development.

**Figure 3:**
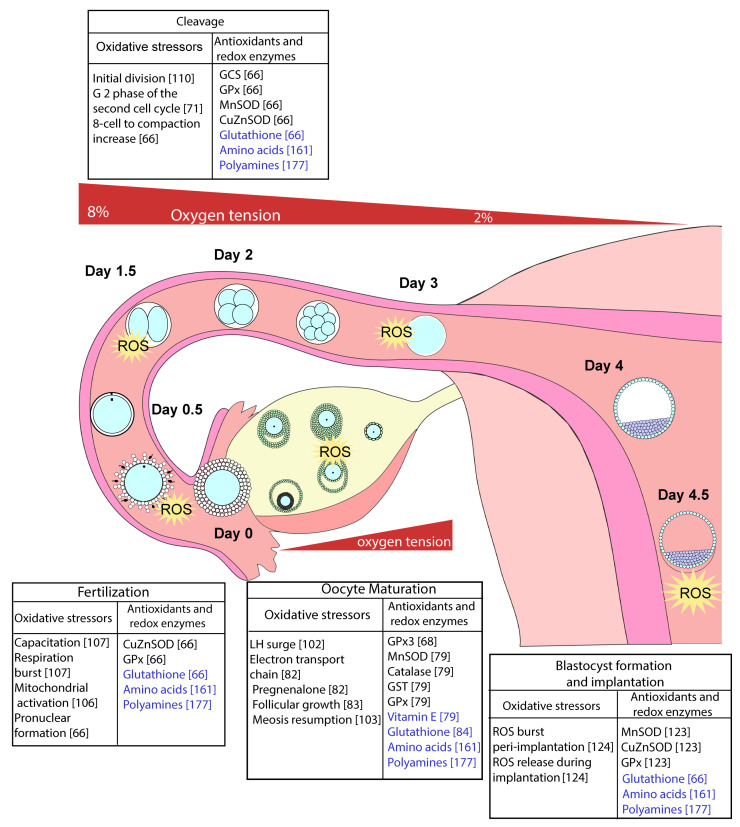
Sources of ROS and antioxidants during *in vivo* embryo development. The preimplantation embryo develops over 5 days in the mouse *in vivo.* The maternal reproductive tract is an environment low in oxygen, with an oxygen gradient of approximately 8% to 2% from the oviduct to the uterus. This, along with a number of antioxidants and antioxidant enzymes, supports redox homeostasis and helps prevent irreversible oxidative damage. During oocyte maturation and preimplantation embryo development, there are numerous oxidative stressors including at ovulation, fertilisation, cellular division, and hatching. Some examples of the control of the action of these stressors by redox enzymes (in black) and antioxidants (in blue) are shown. References are shown in square brackets.

The most common target of ROS and redox-mediated signalling is reversible thiol oxidation of specific redox-sensitive proteinaceous cysteines, generally to form intra- and inter-protein disulphide bonds [41,42]. Many of these signalling mechanisms and targets play homeostatic roles in early development where changes in O_2_ tension and bursts of ROS production are frequent (Figure 3). Oxidative distress, however, can occur, especially during *in vitro* culture of oocytes/embryos where maternal control systems are absent and those of the oocyte/embryo compromised.

These redox-regulated signalling pathways include the NRF2 oxidative stress-response pathway [4]. NRF2 is normally targeted for ubiquitination and proteasomal degradation through its interactions with redox-sensing KEAP1 (the adaptor protein of the Cul3 ubiquitin ligase complex). Oxidant-mediated intermolecular disulphide bond formation between the monomers of the KEAP1 homodimer result in stabilisation of NRF2, which is now free to move to the nucleus and bind antioxidant response elements (AREs). This stimulates expression of a number of genes whose products enzymatically and non-enzymatically reduce the imposed oxidative stress [47].

Analogously, hypoxia-inducible factors (HIF-1α, HIF-2α and HIF-3α) are key transcription factors regulating cellular responses under hypoxic conditions. HIF-α is normally rapidly degraded under high O_2_ tension through hydroxylation of key prolines by the oxidant- and oxygen-sensitive HIF prolyl hydroxylases [48,49]. This targets HIF-α for ubiquitination and subsequent proteasomal degradation. On the other hand, decreased prolyl hydroxylase activity promotes HIF-α stabilisation, movement to the nucleus, binding to hypoxia response elements (HREs), and the upregulation of expression of more than 70 genes, including redox-regulating enzymes, and those which help switch energy demand towards glucose metabolism and away from oxidative phosphorylation [50].

The energy switch away from mitochondria and O_2_ usage under hypoxic conditions can result in the generation of ROS due to the leakage of electrons from the ETC [26]. In a complex interplay, expression of the redox modulator enzyme, Ref-1, is unregulated in the presence of H_2_O_2_. Ref-1 then stimulates gene expression of HIF-1α, which in turn upregulates the expression of HIF prolyl hydroxylases resulting in negative feedback control of HIF-1α activity, even while H_2_O_2_ induces disulphide bond-mediated dimerisation and inactivation of the HIF prolyl hydroxylases [48]. Ref-1 can also reduce disulphide bonds in a number of transcription factors, including HIF-1α, the presence of which generally suppresses their transcriptional activity [51]. The complexity of these homeostatic and interconnected redox-dependent mechanisms, which have been elucidated to some extent in various cellular systems, have been much less explored in oocyte/embryo systems where, nevertheless, O_2_ tension and ROS are known to play major roles.

The FoxO class of transcription factors is a target of redox-mediated signalling and, in turn, a mediator of response to ROS: Growth factor- and signalling-mediated increases in ROS lead to phosphorylation of specific FoxO serines and threonines and result in increased expression of genes whose protein products are responsible for redox regulation, including MnSOD, PRx3 and 5, GPx1, mitochondrial thioredoxin, mitochondrial thioredoxin reductase and catalase. In addition, H_2_O_2_-mediated FoxO activation occurs under conditions of nutrient deprivation and results in upregulation of expression of other key genes including those for autophagy [43,44,45].

#### 2.2.2. Oxidative Distress

Whilst oxidative eustress represents the cellular responses to homeostatic levels of ROS, excessive levels of ROS result in oxidative distress and a range of cellular pathologies, often resulting in growth arrest and apoptosis [4]. In contrast, increased ROS are also associated with a downregulation of tumour suppressor genes and an increase in pro-survival pathways [52,53]. Aberrant upregulation of expression of NADPH oxidase genes can result in various pathologies [54] including those linked to cancer, diabetes, and a number of inflammatory disorders [20,53]. Dysregulation of redox status during oxidative stress can contribute to the formation of metabolic diseases, hinder cellular metabolism, and block cellular antioxidant defence mechanisms [20,55,56].

ROS and Ca^2+^ signalling are interlinked. Ca^2+^ is mainly stored within cells in the ER and release of Ca^2+^ into the cytoplasm is involved in numerous cellular functions. Ca^2+^ release is also directly coupled to Ca^2+^ levels within the mitochondria, due to physical and functional coupling of Ca^2+^ channels in the ER and mitochondria (e.g., IP3R and VDAC, respectively) [57]. An increase in cytosolic Ca^2+^ due to release through IP3R causes an increase in mitochondrial Ca^2+^ which activates oxidative phosphorylation and thus ROS production. Ca^2+^ also activates MnSOD, which helps abate excessive ROS accumulation and maintain ROS homeostasis. Nevertheless, sustained elevation of cytosolic Ca^2+^ due to ROS-induced ER stress can disturb the transfer of Ca^2+^ from ER to mitochondria and cause aberrant mitochondrial metabolism and apoptosis [58]. In addition, oxidative distress causes post-translational modifications of proteins responsible for Ca^2+^ signalling [58]. For example, thiol oxidants and ROS can inhibit functioning of the sarcoplasmic reticulum Ca^2+^-transport ATPase (SERCA), preventing uptake of Ca^2+^ into the ER and restoration of resting cytosolic Ca^2+^ levels [59].

ROS and glucose metabolism are also linked: Skeletal muscle cells exposed to exogenous H_2_O_2_ have increased glucose uptake [60], and the expression of the glucose transporter, GLUT1, is upregulated in L6 muscle cells when exposed to continuous exogenous ROS-generating systems (xanthine/xanthine oxidase and glucose/glucose oxidase) [61]. 

## 3. The Effect of Oxidative Eustress and Distress on Oocyte Maturation, Fertilisation, and Embryo Development

Oocyte maturation, fertilisation and embryo development in the reproductive system take place in a highly complex milieu of factors from the mother and the oocyte/embryo itself. During folliculogenesis there is a steady decline in O_2_ tension in the follicular fluid [62,63] and around the time of ovulation there is a decrease in blood flow to the ovary, thus subjecting the oocyte to decreasing O_2_ tension from the primary follicle stage to the point of ovulation [62,63,64]. There is also a decrease in O_2_ tension from the top of the oviduct (~8%), where fertilisation takes place, to the uterus (~2%), at the time of blastocyst formation and implantation [63,65] (Figure 3). ROS are generated under these conditions, sometimes in acute bursts required for developmental progression but, overall, ROS concentrations are normally maintained within their homeostatic ranges by the maternal environment [11,66,67], the maturing and ovulated oocyte, and pre-implantation embryo [11,66,68,69,70,71].

In stark contrast, culture media for oocyte maturation, fertilisation, and embryo development are extremely simple, consisting of little more than buffers, basic salts, and energy sources (generally a combination of lactate, pyruvate and glucose) [72,73,74]. The inclusion of HEPES buffer in culture media, such as those used for fertilisation, drives production of O_2_^.−^ and, subsequently, production of H_2_O_2_ [75]. Many culture media also contain serum as a protein source for embryo development and/or serum albumin, which reduces adhesion of embryos to surfaces and promotes hatching [76,77]. However, both are a source of ROS generation [11,75,78]. These differences between the *in vivo* and *in vitro* situations present many challenges for assisted reproduction. Some of these challenges and solutions are explored below.

### 3.1. Oocyte Maturation and Fertilisation

The follicular fluid in which oocytes are bathed is rich in antioxidants (including vitamin E (α-tocopherol), β-carotene and GSH) and redox-controlling enzymes (including glutathione peroxidase (GPx) 3, superoxide dismutases (CuZnSOD, MnSOD, SOD3), catalase, glutathione *S*-transferase (GST), and glutathione reductase (GRx) [67,79]. In addition, cumulus cells provide the oocyte with GSH, as well as cysteine and NADPH for generation of GSH, via gap junctions connecting the cells in the follicle [70,80]. This well-balanced redox system maintains homeostatic levels of ROS and an appropriate environment for folliculogenesis and oocyte maturation [67,69,79,81,82,83]. Excess ROS in the follicular fluid results in damage to oocytes, including to the genome and lipid membranes [84]. In addition, the composition of follicular fluid is dynamic during folliculogenesis: As follicles increase in size, total antioxidant capacity (TAC) (a measure of the amount of ROS scavenged by a sample [85]) increases and H_2_O_2_ decreases [84]_._ The increase in TAC appears to be required to combat what would otherwise be a rising concentration of H_2_O_2_ [84]. 

There’s a close correlation between ROS in the follicular fluid on the one hand and oocyte grade on the other [86,87,88]: Follicles for which the follicular fluid has an average ROS level <~70 cps/400 μL produce grade III oocytes, which fertilise and produce grade I and II 4-cell embryos, whereas follicles for which this average is >~85 cps/400 μL produce less mature (grade I and II) oocytes, which generally don’t fertilise and, when they do, produce lower quality (grade III and IV) embryos [88]. An upper control limit of 107 cps/400 μL significantly distinguishes the fertilisation percentage, embryo quality, and the extent of embryo DNA fragmentation regardless of the cause of infertility (surgically removed fallopian tubes, endometriosis or polycystic ovary syndrome) [88], suggesting that high ROS levels represent a generalised cause of failure for *in vitro* fertilisation (IVF) regardless of the underlying physiology. Consistent with this, follicles from IVF patients that have a greater percentage of ROS-producing granulosa cells (77% compared to 61%) are less likely to contain an oocyte [87]. Similarly, blastocysts generated from oocytes where the percentage of ROS-producing granulosa cells are high (70%) do not implant, whereas blastocysts generated from oocytes with only 40% ROS-producing granulosa cells do implant [87].

Whole-body irradiation of female mice in the pre-ovulatory stage results in a 6-fold increase in chromosomal abnormalities in the metaphase plate of fertilised embryos, which can be partially overcome by intraperitoneal injection of vitamin E prior to irradiation [89].

The use of cryopreserved gametes presents challenges for ART. Cryopreserved spermatozoa have decreased antioxidant capacity, which leads to increased ROS in embryos [90,91]. Cryopreservation of oocytes subjects them to oxidative stress and makes them more susceptible to oxidative damage [92,93,94] and increased risk of failure of IVF and intracytoplasmic sperm injection (ICSI) [93,95]. Embryos derived from vitrified oocytes have >1000 differentially expressed genes at the 2-cell stage compared to embryos generated from fresh oocytes. Some of the genes whose expression is altered are related to redox pathways. For example, *GPx6* expression is increased, possibly as a compensatory mechanism to protect against the effects of oxidative stress that occurred during oocyte vitrification [92]. Prolonged incubation of spermatozoa and oocytes during IVF also leads to increased environmental ROS due to its release from immature or dead spermatozoa. To some extent, this can be combatted by decreasing incubation time during fertilisation [96].

During *in vivo* maturation, oocytes are subjected to decreasing O_2_ tension (Figure 3). Despite this, increases in O_2_ consumption and ROS levels are required at critical times to promote further development. For example, O_2_ consumption increases in oocytes as ovulation approaches, coinciding with a switch in energy source from pyruvate to glucose [97,98]. In addition, an increase in the level of H_2_O_2_ (from 66 to 77 ng H_2_O_2_/oocyte) is required for the resumption of meiosis in rat oocytes from the diplotene to MI stage [83]. The burst in H_2_O_2_ modulates signalling by reducing cyclic nucleotide concentrations (cAMP and cGMP) resulting in phosphorylation of CDK1 (at Thr14/15), which in turn destabilises maturation-promoting factor to allow completion of meiosis II [99,100].

Intra-oocyte defence mechanisms during meiotic maturation are important for protecting DNA from oxidative damage. For example, catalase is localised to the nucleus at the germinal vesicle stage and in the peri-chromosomal region following breakdown of the nuclear envelope and thereby protects the DNA from ROS-related damage [101]. The LH surge, which triggers the final stages of oocyte meiotic maturation, induces an increase in ROS, which is required for EGF receptor-mediated signalling events that are essential for maturation. Thus, exogenous antioxidants, such as N-acetylcysteine (NAC) or butylated hydroxyanisole (BHA) prevent LH-induced activation (by phosphorylation) of the EGF receptor and its downstream effector p42/44 MAPK [102]. Similarly, if ROS is reduced *in vitro* using scavengers such as 2(3)-*tert*-butyl-4-hydroxyanisole or nordihydroguaiaretic acid (NDGA), oocyte maturation is also inhibited [83,103]. On the other hand, oocytes with H_2_O_2_ over 90 ng/oocyte undergo apoptosis [83]. These results show that while bursts in ROS are critical for developmental progression, they must still be carefully controlled.

The redox state of oocytes is further altered during fertilisation, with a peak in both O_2_ consumption and ROS [104]. The burst in O_2_·^−^ resulting from increased NADPH oxidase and mitochondrial activity in bovine sperm results in redox-induced efflux of cholesterol from the sperm plasma membrane, and a large global increase in tyrosine phosphorylation driven by cAMP/PKA signalling [105,106,107,108,109]. Furthermore, serum or serum albumin promotes a burst of ROS that triggers capacitation (and subsequent fertilising ability) of sperm at the early, intermediate and late stages through activation of an interconnected panoply of signalling pathways including PKA and ERK, and this can be inhibited by incubation of sperm with antioxidant enzymes such SOD and catalase [78]. 

Similarly, ROS production in sperm-activated bovine oocytes peaks during sperm penetration/sperm-head decondensation, with subsequent peaks at the initiation of pronuclear formation and at the time of the first mitotic division [110]. Again, these bursts in ROS are normally kept under appropriate homeostatic control by the antioxidant defence mechanisms of the oviductal fluid, which includes maternal upregulation of the expression of CuZnSOD and GPx [11,66,111].

Increased ROS commonly leads to male infertility, with high ROS levels causing DNA damage and reducing fertilisation *in vivo* and in IVF [112,113,114]. During ICSI, using sperm with high levels of ROS can still result in successful fertilisation but embryo quality is compromised and the percentage of live births reduced, presumably in part as a result of increased sperm DNA damage [115]. The problem is exacerbated as a result of ROS-containing medium being injected into the oocyte along with the sperm [116].

Female reproductive ageing is tightly linked to a decrease in ovarian antioxidant enzymes and hence an increase in oxidative stress. Granulosa cells in older IVF patients have reduced expression of CuZnSOD, MnSOD and catalase, and morphologically defective mitochondria and ER [117,118]. Aged oocytes show abnormalities in the structure of organelles, consistent with the effects of oxidative stress, including dilated smooth ER and Golgi apparatus, and abnormal mitochondria [118]. Increased oxidative stress during reproductive ageing results in damage to DNA and organelles leading to increased aneuploidy in the oocyte [119,120,121]

### 3.2. The Preimplantation Embryo

ROS levels remain relatively constant during the cleavage stages of *in vivo* pre-implantation development (i.e., 2-cell to 8-cell stages in the mouse) [71] and, following compaction, the embryo increasingly relies on the use of glucose over pyruvate, which may be required to support the proliferative burst and differentiation that begins at this time [122,123].

Peri-implantation blastocysts produce a burst of O_2_^.−^ and decrease levels of SOD compared to pre- and post-implantation embryos [124]. *In vitro*, the addition of O_2_^.−^ around the time of hatching results in thinning of the *zona pellucida*, suggesting that the burst of ROS production for *in vivo* blastocysts assists in hatching. Furthermore, a range of O_2_·^−^ scavengers used on both *in vitro* and *in vivo* blastocysts reduces hatching [124].

**Figure 4:**
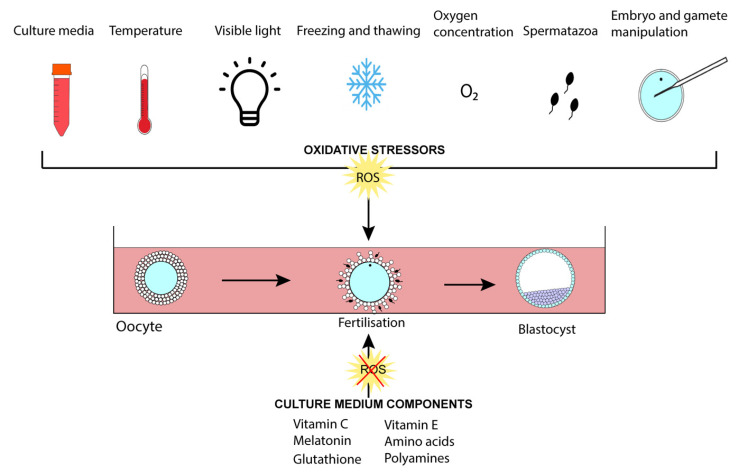
Sources of ROS for *in vitro* cultured oocytes and embryos. Oxidative stress can be induced by a number of laboratory processes, as shown, and a number of antioxidants can be added to the culture medium to combat these excessive ROS levels.

Excessive accumulation of ROS, however, at various times in the life of the preimplantation embryo compromises development, and many problems have been identified that contribute to this in the *in vitro* environment (Figure 4) [125,126]. Among these are: (i) The simplicity of culture media, including especially their generally poor antioxidant properties. (ii) Culture in, or even brief exposure to, 21% O_2_, which results in generation of excess ROS. (iii) Laboratory light, which stimulates ROS production. 

Various strategies have been used to overcome, or at least identify, these problems. For example, TAC assays [85] can be used to quantify the embryo’s ability to cope with oxidative stress. In human IVF cycles, embryos with a higher TAC as measured in culture medium on day 1 had improved fertilisation and cleavage rates, improved development to the blastocyst stage, and less embryonic fragmentation on day 3 of development than those with a lower TAC [125]. *in vitro* cultured embryos have higher levels of aneuploidy than their *in vivo* counterparts and the increased oxidative stress in the laboratory is a contributing factor to this, including culture media components, pH, osmolality, laboratory light and O_2_ concentration [126,127].

The maternal reproductive tract provides support for the developing embryo and, consistent with this, co-culture of mouse embryos *in vitro* with human oviductal epithelial cells improves development: 75, 60, and 40% embryos develop to the 4-8 cell, blastocyst stage and hatching stage, respectively, compared to 20, 3, and 0% in contactless coculture or without coculture [128]. Coculture eliminates the build-up of O_2_·^−^ in the medium [128]. In a related study, maternal expression of oviductal microsomal epoxide hydrolase (*Ephx1*), an enzyme important in detoxifying genotoxic molecules, is upregulated in the oviduct over the first 5 days of mouse embryo development [129]. In addition, inhibition of human EPHX1 by cyclohexene oxide or 1,1,1-trichloropropene-2,3-oxide in human oviductal cells cocultured with mouse embryos increases ROS and prevents the beneficial effect of co-culture on blastocyst formation and hatching [129].

Knockout of very few redox-regulating/antioxidant genes pose issues for preimplantation development [23] presumably, in part, due to mechanistic redundancy. However, mouse embryos knocked out for *Trx1*, the gene for the small antioxidant protein Trx, are embryonic lethal at ~E3.5 [130] and knockout of the redox-modulating enzyme, Ref-1 [48,51], is embryonic lethal shortly after implantation [131]. Knockout/knockdown of some redox-sensing/regulating transcription factors can also disrupt development at an early stage. Knockdown of FoxO proteins (FoxO1_,_ FoxO3 and FoxO4) in mice impairs preimplantation embryo development [132]: The majority of the FoxO knockdown embryos arrest at the 2-cell stage, and blastocyst formation decreases from ~70% to ~25%. ROS in these embryos is elevated 3–4 fold, and in the 2-cell arrested embryos there are 1.5–3 fold increases in Fasl and cleaved caspase 3 (responsible for apoptosis) and p53 and p21 (responsible for cell-cycle arrest) [132].

#### 3.2.1. Use of Antioxidants for *In Vitro* Culture

In embryo culture media, usually the only antioxidant included is a chelator, such as EDTA, which sequesters redox catalysts such as heavy metal ions. In particular, H_2_O_2_ and O_2_·^−^ can react in the Fe^2+^/Fe^3+^ Haber-Weiss catalytic cycle to form the highly reactive OH· [11,133], which in many species is responsible for preimplantation block [71,134,135]. In mouse, the addition of EDTA to culture medium overcomes the 2-cell block (which occurs at the G2 phase of the cell cycle) predominantly by acting as a chelator [134,136]. Consistent with this, a 30-min exposure of mouse embryos *in vitro* to exogenously added H_2_O_2_ (50 μM) reduces the percentage that pass the block from >70% to 40% [137,138], while addition of the reducing agent *N*-acetyl-L-cysteine along with H_2_O_2_ completely overcomes this [137,138].

The addition of GSH to mouse embryo culture medium reduces ROS in these embryos and increases the percentage of blastocysts [139]. Similar results were obtained in the porcine [140] and bovine systems [141]. Consistent with the importance of GSH, knockout mice for GPx4 [142], γ-glutamylcysteine ligase [143] or glutathione synthetase [144], all of which are required for the formation of GSH, are embryonic lethal.

Melatonin, a tryptophan-derived hormone secreted from the pineal gland to regulate circadian rhythm, is also an antioxidant that scavenges a number of ROS, including OH· and H_2_O_2_, and also upregulates the expression of GPx, catalase, SODs and glutathione reductase [145]. Melatonin improves viability of heat-stressed bovine embryos in culture [146]. Oocyte retrievals in women treated with melatonin had, on average, double the fertilisation rate compared to previous cycles, while simultaneously having a one-third reduction in the concentration of intrafollicular concentration of 8-hydroxy-2′-deoxyguanosine (8-OHdG), a biomarker of oxidative stress [89,147,148]. The inclusion of melatonin in culture medium for embryo development reduces cleavage times, improves development to the blastocyst stage, and decreases ROS in vitrified embryos [94,149]. 

Similarly, vitamin C (ascorbic acid) and vitamin E scavenge ROS, and culture medium for porcine embryos supplemented with vitamins C or E reduces the toxic effects of culture under 21% O_2_, increases blastocyst cell numbers, and the percentage of embryos that develop to the blastocyst stage, though there is a complex dependence on concentration of the antioxidants and supplementation timing [150,151]. The addition of vitamin C to culture medium enables embryos to develop to the blastocyst stage even when they are exposed to oxidative stress by incubating them with PMA-activated leukocyte supernatant [152].

In addition to oxidative stress linked to mitochondria and the cytosol, significant effects can also occur in the ER. ROS accumulation in the embryo can activate the unfolded protein response (UPR) pathways leading to accumulation of unfolded or misfolded proteins in the ER, apoptosis, changes in gene expression, and developmental errors [153,154]. Mouse embryos cultured in the presence of 2% DMSO, which causes oxidative stress, have increased mitochondrial Ca^2+^, resulting in mitochondrial-dependent apoptosis [155]. In addition, the morula cultured in 2% DMSO upregulate expression of ER stress genes *GRP78/BIP* and UPR genes including *Hspa5*, *Hsp90b1*, *Ddit3*, and *Atf4* which contribute to the increased apoptosis and developmental arrest at the 2-cell, 4-cell, and morula stages in a dose-dependent manner [155]. Reducing ER stress in cultured bovine embryos using the bile-acid tauroursodeoxycholate decreases ROS and increases the percentage of embryos that reach the blastocyst stage [156].

A large number of other antioxidants from a variety of sources have been used to promote oocyte and embryo development *in vitro*, generally with similar success, although determination of the appropriate concentration range for beneficial effect is an issue [146,157,158,159,160]. 

#### 3.2.2. Amino Acids and Polyamines

The addition of individual and combinations of proteinogenic and other amino acids to culture media is also commonly used due to the presence of amino acids in the oviductal and uterine fluids [161,162,163,164]. Of these, proline and its close analog pipecolic acid can reduce ROS by a number of possible mechanisms, including: (i) Direct scavenging by the secondary amine of the ring structure [32,165,166]. (ii) Metabolism to glutamate, a precursor for GSH production [35,167,168]. (iii) Suppression of ETC activity (a major cellular source of ROS) [29,169,170]. In this last case, the suppression by proline involves POX, the enzyme that converts proline to P5C [33,34]. While this initially results in acute generation of ROS (due to the physical coupling of POX to Complex II) [29], the long-term effect is downregulation of the expression of ETC genes, and hence the activity of the ETC [29].

Proline acts as a cryoprotectant for oocytes [93] and sperm [171] in part by acting as a ROS scavenger, which reduces the damage associated with oxidative stress. Proline added to the storage medium for boar sperm also results in increased GSH, and catalase and SOD activity. Upon thawing, sperm parameters improve, including those for motility and acrosome integrity. Oxidative stress upon exposure of thawed semen to H_2_O_2_ is also reduced in the presence of L-proline [171].

The addition of proline or pipecolic acid only during fertilisation reduces mitochondrial activity by 40% and ROS levels by 60%, and improves later development to the blastocyst stage and hatching [162], consistent with the idea that a metabolically ‘quiet’ oocyte has better developmental potential. Similarly, proline added during embryo culture also improves development to the blastocyst stage [163]. In the porcine trophectoderm cell line, pTr, proline reduces ROS, the mRNA expressions of glutamate-cysteine ligase catalytic subunit and glutathione synthetase increases, as do GSH levels [169]. Selected other amino acids can sometimes substitute for proline: For example, glutamine and hypotaurine reduce H_2_O_2_ levels in porcine embryos, improve blastocyst development and blastocyst cell numbers, and reduce DNA damage [172]. Though the molecular mechanisms are unclear, those for glutamine (a precursor for glutathione) don’t appear to be glutathione dependent, and it’s notable that the antioxidant, hypotaurine, can’t be synthesised by oocytes/embryos and therefore would normally be supplied from maternal sources [172,173].

However, the effects of amino-acid supplementation of oocyte/embryo culture medium are not easily predictable: While some amino acids are beneficial to maturation/development, other amino acids inhibit their beneficial effects (e.g., by competing for uptake via amino-acid transporters), or are themselves toxic [162,163,174,175]. For example, supplementation of IVF culture medium with glycine, cysteine and glutamate, the three amino acids required for producing GSH, reduce development of bovine embryos [174]. 

Polyamines, such as spermine and spermidine, can scavenge H_2_O_2_-generated OH·, via the Fenton reaction, as well as ^1^O_2_ [176] and are essential components of seminal fluid and the female reproductive tract; their absence resulting in male infertility and failed embryogenesis [177]. The addition of 25 µM spermine or spermidine to culture medium containing high glucose, a condition that induces oxidative stress, reduces lipoperoxidation in mid-gestation rat embryos and reverses developmental defects [178]. Spermine (10–500 µM) increases GSH concentrations and decreases ROS in *in vitro* matured porcine oocytes and increases the percentage of blastocysts following parthenogenetic activation [179]. Given that proline can be a major source for the production of polyamines [177,180,181], these data are suggestive of an additional role of this amino acid in developmental processes. Consistent with this, the porcine placenta produces polyamines using proline as the major amino-acid source [181].

#### 3.2.3. O_2_ Tension and Oxidative Stress

21% O_2_ imposes oxidative stress on embryos, as measured by ROS accumulation and its effects, as well as triggering mechanisms which attempt to reduce the stress. For example, bovine embryos cultured at 21% O_2_ have fewer inner cell mass (ICM) and trophectoderm (TE) cells than those cultured at 5% O_2_, despite upregulation of expression of oxidant-reducing pathways, such as the NRF2 pathway, and a range of antioxidant enzymes [182,183].

*In vitro* culture of embryos alters mitochondrial structure, with O_2_ tension affecting the extent of this change [184]. Blastocysts developed in 21% O_2_ have more abnormal mitochondria, with more mitochondrial vacuoles and less mitochondrial DNA, compared to blastocysts flushed from the uterus [184]. These problems are reduced in 5% O_2_ [184]. Similarly, mitochondrial activity in bovine blastocysts improves if O_2_ tension is reduced from 21% to 5% [183]. Consistent with this, addition of 30 μM H_2_O_2_ to culture medium of mouse zygotes to induce oxidative stress reduces mitochondrial membrane potential, and mitochondrial activity declines by 40% [185]. 

Compared to bovine embryos cultured in 5% O_2_, those cultured in 21% O_2_ have increased ROS production. 21% O_2_ activates the NRF2 redox-sensitive stress-response pathway from the 8-cell stage through to the blastocyst stage: The expression of *NRF2* increases 2-4 fold at these stages while that of the NRF2 inhibitor, *KEAP1*, halves [183]. Consistent with this, the expression of several NRF2-responsive antioxidant genes such as *SOD1* (*CuZnSOD*) and *PRDX1* increase. Nuclear localisation of NRF2 also increases in blastocysts formed at day 7, consistent with its role as a transcription factor binding to chromosomal antioxidant response elements [183].

High O_2_ tension and oxidative stress in cultured embryos can reduce developmental potential, result in DNA fragmentation, modifies DNA methylation patterns and histones, and the expression of redox-sensitive genes [182,186]. For example, bovine oocyte culture in 21% O_2_ leads to downregulation of expression of *PAF1* and *REST* which are important in chromatin organisation and histone modifications as well as for maintaining a state of pluripotency [182]. Oxidative stress also leads to an upregulation of *SOX2* and *HP1*, both of which are involved in changes to DNA methylation and chromatin remodelling [182]. These changes indicate that oxidative stress can alter the epigenetic landscape and interfere with embryo development [187]. 

Similarly, there are a large number of gene expression changes (≥2-fold) between embryos cultured in 21% O_2_ compared to those cultured at 2–5% O_2_ [188,189]. These include genes involved in numerous critical pathways, including biosynthesis, mitochondrial activity, kinase activity, and the microtubule-based cytoskeleton [189].

Since culture in 21% O_2_ can induce oxidative stress [190,191], some ART laboratories culture in 5% O_2_ [192], which improves mitochondrial membrane potential and upregulates the expression of genes encoding for antioxidant enzymes such as MnSOD and PRDx5 [193]. However, even transient exposure to 21% O_2_ at any time during assisted reproduction can compromise the embryo. For example, oocyte culture in 5% O_2_ results in higher rates of fertilisation compared to those incubated in 21% O_2_ [194] but switches in O_2_ tension and even temporary removal of oocytes and embryos from low O_2_ tension to atmospheric causes changes sufficient to induce oxidative stress [194,195]. For example, exposure of mouse zygotes to atmospheric O_2_ for 1–2 h reduces the percentage of embryos that reach the blastocyst stage by a third to a half [196].

#### 3.2.4. Laboratory Light

Mouse and hamster zygotes exposed to laboratory light for as little as 15 min have increased H_2_O_2_ and a variety of developmental deficiencies ensue including, in the mouse, an increased percentage of apoptotic cells in the blastocyst and a reduction in live births and, in the hamster, complete cessation of development to the morula stage [197]. Embryos exposed to white light also have lower implantation capacity and a higher percentage of apoptosis and DNA fragmentation, both of which are associated with oxidative stress [198]. Minimising laboratory light poses challenges when performing procedures and monitoring embryo development during IVF and many other forms of ART [197,199]. 

## 4. Transgenerational Effects of ROS

The developmental origins of health and disease hypothesis (DoHaD) states that inappropriate cues in the embryonic environment can result in cellular reprogramming and transcriptional changes, causing disease in offspring up to an including adulthood, and these transcriptional changes potentially have transgenerational effects [200]. The suboptimal environment in which oocytes/embryos are cultured can result in oxidative distress having immediate impact not only on the success rates of assisted reproduction but also long-term effects on fetal, child and adult health and, potentially, the offspring of future generations.

Consistent with this, there is an increase in epigenetic anomalies in children born via ART, some of which are linked to epigenetic changes and imprinting errors. The lack of antioxidants, and enzyme-mediated antioxidant control, in oocyte/embryo culture media can result in ROS-mediated oxidation of methylcytosine, the necessary precursor to active demethylation of DNA at some CpG islands [201]. These immediate and aberrant ROS-mediated epigenetic modifications can result in changes to gene expression as well as long-term, including transgenerational, changes [13,14] such as large-offspring syndrome, enlarged organs and metabolic disorders [13].

Cryopreservation exacerbates ROS production in oocytes [93]. In the mouse, the first filial generation offspring derived from cryopreserved oocytes compared to fresh oocytes have increased diastolic blood pressure and increased triglyceride levels as adult mice [12]. Oocytes cryopreserved in the presence of proline have decrease in these transgenerational oxidative stress responses. The mechanisms have not been investigated but proline can act by a number of possible ROS-reducing mechanisms, as outlined above [29,33,34,165,166,167,168,202,203]

The endocrine-disruptor, bisphenol A (BPA), can disrupt development via a number of mechanisms including increasing ROS production, altering embryo metabolism and mediating epigenetic modifications. Exposure of bovine oocytes to BPA causes an increase in ROS, decreases oocyte maturation, increases DNA damage and increases histone modifications [204]. Oocytes exposed to BPA in the F0 generation can result in behavioural and other phenotype changes for up to 3 generations [205]. 

High levels of ROS in sperm can cause epigenetic changes. Obesity results in poorer sperm quality and an increase in spermatic ROS leading to epigenetic changes in the sperm that may be the cause of acquired obesity in future generations [206]. 

As oxidative stress intensifies, the genetic and epigenetic effects on the gametes increase: Exposure to low levels of oxidative stress causes oxidation of bases and the generation of abasic sites whereas more intense oxidative stress can result in DNA strand breaks [207]. These DNA strand breaks and changes pose a mutagenic risk to the gamete and changes in the nucleus and to DNA can result in mutations in future generations [207]. 

Collectively, the deficiencies of oocyte/embryo culture media and their inability to properly support protection from oxidative stress, as would normally occur *in vivo*, results in more frequent changes to the epigenetic landscape [201,208], an increase in the number of offspring with genetic imprinting anomalies, and therefore increased likelihood of transgenerational effects in children and animals born using ART. 

## 5. Emerging Therapies and Trends in ART

Some of the relationships between oxidative stress and antioxidants have been fairly well established in animal models including the testing of oral supplementation of antioxidants to reduce ROS levels in follicular and seminal fluids [93,209,210,211,212,213,214,215,216,217,218,219,220,221,222,223,224,225]. Based on this, there have been a number of human clinical trials testing various antioxidants—e.g., melatonin, myoinositol, coenzyme Q10, and multivitamin combinations (principally vitamins C and E)—and their effects on outcomes of assisted reproduction (Table 1). 

These listed trials were carried out with patients undergoing fertility treatments, including IVF and ICSI. They aimed to reduce oxidative stress in gametes and embryos, as measured by a number of parameters including fertilisation rate, embryo quality, clinical pregnancy rates, sperm motility and morphology, as well as antioxidant measures including TAC, lipid peroxidation (LPO) and antioxidant enzyme levels. Overall, oral supplementation with antioxidants improves the fertilisation rates, embryo quality, and pregnancy rate (Table 1). 

Clinical trials are also being carried out testing the exogenous addition of antioxidants to media for sperm, oocyte maturation, fertilisation and embryo culture and their impact on gametes/embryos (Table 2). They aimed to reduce oxidative stress as measured by a number of parameters, including sperm quality, fertilisation rate, embryo development and blastocyst formation. Overall, addition of antioxidants to media results in improved gamete quality and an increase in clinical pregnancies (Table 2). 

Given that 21% (atmospheric) O_2_ results in increased ROS, many IVF clinics have switched to low-O_2_ incubators to more closely mimic O_2_ concentrations of 2–8% in the reproductive tract [63,65]. Several clinical trials have been carried out to determine the impact of low concentrations of O_2_ on various parameters of fertility [226,227,228]. Culture of embryos in 5% O_2_ increases the percentage developing to the blastocyst stage and their quality. However, there is limited evidence to show low O_2_ improves live-birth rate [226,227,228]. 

**Table 1:** Clinical trials using antioxidants, taken orally, in patients undergoing infertility treatment, and their effect on oocyte, sperm, and embryo health.

Antioxidant	Trial Type	Population	Method	Results	Reference
Melatonin	Retrospective	Women with poor oocyte quality or low embryo quality in previous cycles.	3 mg/day oral melatonin for ≥2 weeks until the day of hCG trigger dose.	Improved fertilisation rates and improved embryo quality. No effect on oocyte maturation or percentage of blastocyst development.	[209]
Melatonin	Randomised clinical trial	Women 20–45 years undergoing IVF.	3 mg/day oral melatonin from the day of GnRH antagonist until the day of embryo transfer.	Increased percentage of mature oocytes and grade 1 embryos. No effect on pregnancy rates	[210]
Melatonin	Randomised pilot study	Women with unexplained infertility undergoing a second IVF cycle.	Groups allocated to 0, 3, or 6 mg/day oral melatonin from first appointment to start of ovarian stimulation (i.e., 40 days).	Both doses of melatonin increased levels of melatonin, TAC, and lipid peroxidation in follicular fluid; 6 mg/day melatonin increased SOD. Both 3 and 6 mg/day melatonin increased the number of oocytes retrieved, fertilisation percentage, and number of transferable embryos.	[211]
Myoinositol and melatonin	Randomised double-blind clinical trial	Women with PCOS undergoing IVF treatment.	Women were allocated to the following groups: Control, 4 g myoinositol or 4 g myoinositol + 3 mg melatonin, orally twice per day, from cycle day 1 to 14 days post embryo transfer.	Melatonin increased the percentage of mature oocytes and number of high-grade embryos. No effect on pregnancy rate.	[212]
Myoinositol and melatonin	Prospective clinical trial	Women aged 30–40 with one or more unsuccessful IVF cycles due to poor oocyte quality.	Daily oral supplementation with 4 g myoinositol + 1.8 mg melatonin for 3 months prior to IVF cycle.	Increased number of mature oocytes. No effect on the percentage of mature oocytes, fertilised embryos, or grade of embryos.	[213]
Coenzyme Q10	Randomised control trial	Women aged <35, with poor ovarian response to stimulation undergoing IVF/ICSI.	Oral administration of 200 mg CoQ10 3 times per day for 60 days prior to IVF/ICSI cycle.	Decreased day 3 FSH, increased peak E2 concentration, number of oocytes retrieved, fertilisation rate, and embryo quality. No effect on clinical pregnancy rate.	[214]
Coenzyme Q10	Controlled clinical study	Women undergoing IVF-ET for unexplained or tubal disease-related infertility.	Oral supplementation of 200 mg CoQ10 daily for 30 days before oocyte pick up.	Increased follicular fluid CoQ10 in its reduced form. Decreased TAC in patients aged >35 years.	[215]
Coenzyme Q10	Retrospective study	Women with poor ovarian reserve undergoing IUI or IVF cycles.	Daily oral administration of either 75 mg DHEA alone or 75 mg DHEA + 600 mg CoQ10.	Improved ovarian responsiveness with an increase in antral follicular count and number of mature follicles. No change in blastocyst development or pregnancy rates.	[216]
Growth hormone	Randomised control clinical trial	Women with poor ovarian reserve undergoing IVF.	4 IU/day growth hormone injected subcutaneously from day 2 of the previous menstrual cycle until trigger day (36–48 days).	Increased endometrial thickness, implantation rate, and clinical pregnancy. Increased TAC, decreased total oxidative stress index in follicular fluid. Decreased ROS in granulosa cells. Increased embryo quality, implantation rate, and clinical pregnancies.	[217]
N-acetyl cysteine (NAC)	Placebo-controlled double-blind, randomised clinical trial	Women with PCOS undergoing IVF.	Oral administration of 1.2 g NAC on days 3–7 of the menstrual cycle.	Increased number of follicles, ovulation rate, pregnancy rate, and endometrial thickness.	[218]
Pentoxifylline and vitamin E	Randomised clinical trial	Women <39 years of age with various forms of infertility undergoing ICSI-ZIFT.	Daily oral administration of 400 mg vitamin E and 400 mg pentoxifylline for two cycles before ZIFT.	Improved clinical pregnancy rate.	[224,229]
Multivitamin and mineral	Controlled clinical trial	Women undergoing infertility treatment.	Oral multivitamin and mineral supplementation for 45 days before oocyte collection.	Decreased lipid peroxidase levels in follicular fluid and serum. Increased GSH and vitamins C and E in follicular fluid.	[219]
FertiMax2 (Vitamins C and E, zinc, selenium, L-carnitine, folic acid, and coenzyme Q10)	Preliminary clinical study	Males with male factor infertility undergoing IVF/ICSI.	Oral administration of Fertimax2 for 2–5 months prior to partner’s IVF/ICSI cycle.	Increased fertilisation, cleavage, embryo quality, implantation, and clinical pregnancy rate. No effect on semen parameters.	[220]
Menevit (Lycopene, vitamins C and E, zinc, selenium, folate, garlic oil)	Retrospective cohort analysis	Males with male factor infertility undergoing IVF/ICSI.	Single daily capsule for an unrecorded amount of time.	Increased clinical pregnancy and live birth rate.	[225]
Micronutrient antioxidants (Vitamins, folates and minerals)	Preliminary study	Women aged >39 years undergoing infertility treatments with one failed embryo transfer.	After one typical GnRH antagonist cycle, failed patients were prescribed a daily capsule of micronutrient antioxidants for three months before embryo transfer.	Increased TAC and free thiol availability in follicular fluid and serum. Decreased number of poor grade embryos. No change in fertilisation or cleavage rates.	[222]
Mixed antioxidant formulation (Vitamins C and E, selenium, L-carnitine, zinc, folic acid, lycopene)	Randomised controlled trial	Males with either low sperm concentration, motility, morphology or high DNA fragmentation.	Allocated antioxidant or placebo for 3–6 months. Semen parameters including concentration, motility, morphology, and DNA fragmentation measured.	Decreased sperm concentration, total sperm count, and total motile sperm. No change in morphology, motility, or DNA fragmentation. No change in pregnancy/live birth rates.	[223]

**Table 2:** Clinical trials using antioxidants in vitro and their effect on oocyte, sperm, and embryo health.

Antioxidant	Trial Type	Population	Method	Results	Reference
Mixed antioxidant formulation (acetyl-L-carnitine, N-acetyl cysteine, alpha-lipoic acid)	Blinded randomised control sibling oocyte study	IVF/ICSI patients under 40 years undergoing fertility treatment.	Formulation added to G-series medium, including 10 μM acetyl-L-carnitine, 10 μM N-acetylcysteine, and 5 μM alpha-lipoic acid added to both fertilisation and culture media.	No effect on fertilisation. Increased percentage of good quality embryos on day 3 (patients <35 years). Increased number of patients (35–40 years) receiving a positive pregnancy test, increased percentage with fetal heart beat and ongoing pregnancy.	[230]
L-carnitine	Retrospective clinical trial	Patients <40 years undergoing infertility treatment.	1 mM L-carnitine added to embryo culture medium from day 1 to day 6.	No effect on percentage of embryos developed to blastocyst stage but increased percentage of good quality embryos on days 2, 3, and 5. Increased blastocyst ICM and TE cell numbers and increased clinical and ongoing pregnancies.	[231]
Coenzyme Q10	Randomised clinical trial	Women 38–46 years and ≤30 years undergoing IVF.	GV stage oocytes randomly allocated to no treatment or antioxidant treatment where oocytes were cultured ±50 μM CoQ10 for 24 h.	For patients 38–46 years, CoQ10 increased oocyte maturation and decreased oocyte aneuploidy. No effect on oocyte maturation or aneuploidy in ≤30 years group.	[232]
Mixed antioxidants (L-carnitine, taurine vitamin B5, vitamin C; other vitamins that are not antioxidants were also added to this formulation)	Non-interventional sibling oocyte study	Women ≤42 years old undergoing ICSI cycles.	Oocytes randomly allocated to medium containing mixed antioxidants or standard continuous single culture medium.	Antioxidant containing medium had no effect on blastulation but showed slower compaction and blastulation rates, and blastocysts were of poorer quality.	[233]
Alpha-lipoic acid	Randomised clinical trial	Normozoospermic men undergoing IVF/ICSI cycles.	Semen samples were randomly allocated to sperm wash medium ±0.02 mM alpha-lipoic acid during centrifugation and incubation for 1 h.	Sperm viability and motility increased while DNA damage and ROS decreased when prepared in wash medium containing alpha-lipoic acid.	[234]
L-carnitine	Randomised clinical trial	Infertile men with normospermia or asthenozoospermia.	Cryopreservation of semen samples in medium containing 1 g/L L-carnitine.	Improved sperm parameters after thawing including motility and viability as well as decreased DNA fragmentation.	[235]

## 6. Conclusions

This review highlights that ROS and oxidative eustress contribute to normal cellular homeostasis, with ROS playing direct and indirect roles in a very wide range of physiological processes. In keeping with this, homeostatic levels of ROS, including timed bursts, are necessary for normal oocyte maturation, fertilisation and embryo development. However, excess ROS production results in tipping the redox balance from eustress to distress, leaving oocytes and embryos susceptible to damage, particularly in the *in vitro* environment where protective maternal factors are absent. Highly simplified culture media, and non-physiological culture conditions (including high oxygen tension and exposure to laboratory light) contribute to reduced success for ART. Oxidative distress disrupts metabolic and signalling pathways, alters the expression of wide range of genes, and changes the epigenetic landscape. These disruptions not only affect the oocyte/embryo but can have transgenerational impacts. 

Animal models and clinical trials show that judicious exogenous addition of antioxidants to culture media or oral supplementation to diet can mitigate the impact of ROS and improve assisted reproduction outcomes. Similarly, the addition of antioxidants to *in vitro* media for gamete preparation and cryopreservation can be beneficial to future embryo development. Nevertheless, the extraordinary complexity of the redox circuitry *in vivo* means much remains to be understood and applied to improve the success in ART.

## Data Availability

No new data were created or analysed in this study. Data sharing is not applicable to this article.
